# Development of an accurate lateral flow immunoassay for PEDV detection in swine fecal samples with a filter pad design

**DOI:** 10.1186/s44149-021-00029-1

**Published:** 2021-11-08

**Authors:** Siyi Zou, Lei Wu, Gan Li, Juan Wang, Dongni Cao, Tao Xu, Aiqing Jia, Yong Tang

**Affiliations:** 1 Department of Bioengineering, Guangdong Province Engineering Research Center of Antibody Drug and Immunoassay, Jinan University, Guangzhou, 510632 China; 2 Guangdong Haid Institute of Animal Husbandry & Veterinary, Guangzhou, 511400 China

**Keywords:** Porcine epidemic diarrhea virus, Latex beads, Lateral flow immunoassay, Sample pretreatment, Filter pad

## Abstract

Porcine epidemic diarrhea virus (PEDV), as the main causative pathogen of viral diarrhea in pigs, has been reported to result in high morbidity and mortality in neonatal piglets and cause significant economic losses to the swine industry. Rapid diagnosis methods are essential for preventing outbreaks and transmission of this disease. In this study, a paper-based lateral flow immunoassay for the rapid diagnosis of PEDV in swine fecal samples was developed using stable color-rich latex beads as the label. Under optimal conditions, the newly developed latex bead-based lateral flow immunoassay (LBs-LFIA) attained a limit of detection (LOD) as low as 10^3.60^ TCID_50_/mL and no cross-reactivity with other related swine viruses. To solve swine feces impurity interference, by adding a filtration unit design of LFIA without an additional pretreatment procedure, the LBs-LFIA gave good agreement (92.59%) with RT-PCR results in the analysis of clinical swine fecal samples (*n* = 108), which was more accurate than previously reported colloidal gold LFIA (74.07%) and fluorescent LFIA (86.67%). Moreover, LBs-LFIA showed sufficient accuracy (coefficient of variance [CV] < 15%) and stable (room temperature storage life > 56 days) performance for PEDV detection, which is promising for on-site analysis and user-driven testing in pig production system.

**Supplementary Information:**

The online version contains supplementary material available at 10.1186/s44149-021-00029-1.

## Introduction

The rapid diagnosis of pathogenic microorganisms is essential to identify diseases and provide the correct preventive medicine or treatment (Carter et al. 2020; Wang et al. 2020a). For animal diseases, the predominant trend is the development of pragmatic means to monitor animal health, with a greater focus on preventive medicine rather than treatment after a disease is contracted. Porcine epidemic diarrhea virus (PEDV), of the genus *Alphacoronavirus* in the family *Coronaviridae*, causes watery diarrhea, vomiting and dehydration, and has 100% morbidity and approximately 80-100% mortality in neonatal piglets (Jung et al. 2020). As an acute and highly contagious enteric disease, porcine epidemic diarrhea (PED) caused by PEDV was first reported in England and Belgium, and the emergence and re-emergence of PED outbreaks have occurred in Europe, America and Asia in recent decades (Diep et al. 2018; Pensaert and de Bouck 1978; Sun et al. 2012; Wood 1977). In particular, PED spread quickly across China after the first outbreak at the end of 2010, causing enormous economic losses and a destructive blow to the pig industry. The threat posed by PEDV still persists (Chen et al. 2019; Gao et al. 2013).

In addition, owing to the indistinguishable clinical symptoms from the pathological and epidemiological changes caused by porcine enteric coronaviruses, such as the closely related coronaviruses (Luo et al. 2020; Malbec et al. 2020; Saif et al. 2019), transmissible gastroenteritis virus (TGEV), and porcine deltacoronavirus (PDCoV), rapid and accurate diagnostic tools are very important for the prevention and control of the spread of PEDV in pigs.

Currently, confirmative detection of PEDV is performed in laboratories by virus isolation, immunofluorescence assay (IFA), enzyme-linked immunosorbent assay (ELISA), quantitative real-time-reverse transcription-polymerase chain reaction (qRT-PCR), conventional RT-PCR, reverse transcription loop-mediated isothermal amplification (RT-LAMP), and reverse transcription recombinase polymerase amplification assay (RT-RPA) (Diel et al. 2016; Fan et al. 2020; Ishikawa et al. 1997; Pan et al. 2012; Ren and Li 2011; Wang et al. 2018; Wang et al. 2020b). Although these techniques provide accurate results, they require technical expertise and specialist equipment; moreover, the processes are cumbersome, making them unsuitable for field use and the rapid management of emergent PED outbreaks.

In contrast to genetic material-based techniques, immunological biosensing diagnostic tools may provide cost-effective diagnosis in primary health care units. Paper-based lateral flow assays (LFAs), an ideal platform for performing immunoassays in a low-cost, easy-to-use manner, have widespread use for on-site screening of diseases in nonlaboratory settings and self-testing by inexperienced pig farmers (Jiang and Lillehoj 2021; Liu et al. 2021; Natarajan et al. 2019; Nguyen et al. 2020; Parolo et al. 2020). Previously, our laboratory developed a sensitive EuNP-based fluorescent LFIA (lateral flow immunoassay) for PEDV detection, but this required an immunofluorescent analyzer to obtain results. Colloidal gold LFIAs can be read by the naked eye but have obvious drawbacks, such as limited analytical sensitivity and batch-to-batch differences in particle size (Bian et al. 2019; Huang et al. 2021a; Zhu et al. 2019). Hence, to overcome these limitations, we chose color-rich dyed latex beads (LBs) as a base for a sensitive colorimetric LFIA that could be read by the naked eye, making use of the exceptional color, brilliance, and resistance of LBs to chemical and physical damage.

At present, the identification of PEDV is mainly through the collection and detection of pig serum, pig intestine contents or pig feces. To the best of our knowledge, the fecal-oral route is believed to be the primary mode of PEDV transmission (Li et al. 2018; Lin et al. 2016; Yuan et al. 2021). Moreover, virions in feces are frequently indirectly transmitted within and between pigpens *via* transport trailers, farm workers’ hands, boots and clothes (Jung et al. 2020). Hence, feces are considered a rapidly obtainable and noninvasive biological sample that could be applied for PEDV detection in veterinary practice. In this study, we developed an LB-LFIA suitable for the detection of PEDV antigens in swine feces.

As shown in our preliminary study, solid residues in swine fecal samples remained on the lateral flow pad, reducing the accuracy of paper-based immunoassays. Thus, a sample pretreatment procedure played an important role in the detection of LFIA in clinical samples. Commonly, magnetic separation, centrifugation and electrophoresis are widely used for the separation and enrichment of targets from complex samples. Moreover, ultrasound actuation with swarming or assembly behavior has also been applied as a pretreatment method for lateral flow biosensors. However, those pretreatment procedures were limited by the need for appropriate equipment (Huang et al. 2021b; Parolo et al. 2020; Tsai et al. 2018). In this study, we integrated membrane filtration and an LFIA platform to achieve sample pretreatment without additional operation. A filtration unit was applied to improve the analytical performance of LBs-LFIA for the analysis of swine feces and was found to yield good agreement (92.59%) with RT-PCR results, which was much higher than that of the reported colloidal gold LFAs (74.07%)(Bian et al. 2019) and fluorescent LFAs (86.67%)(Xu et al. 2020). These results indicate that the LBs-LFIA is sensitive, specific and allowed on-site user-operated detection of PEDV, which could shorten the response time for dealing with potential disease outbreaks.

## Results

### Principle of the LBs-LFIA for PEDV detection

**Fig. 1:**
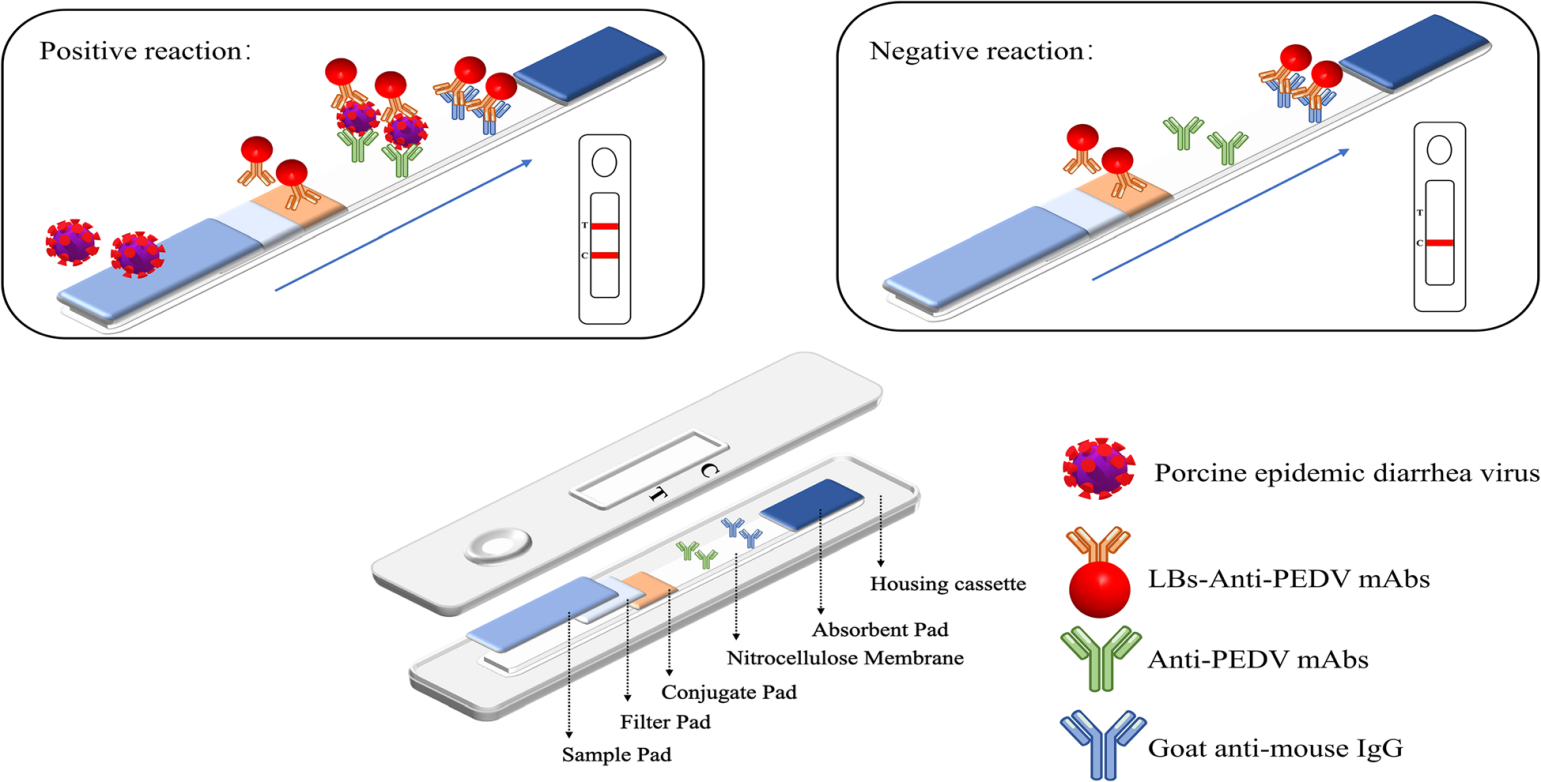
Schematic of PEDV detection using the LBs-LFIA. *PEDV*, porcine epidemic diarrhea virus

Once the viral analyte reaches the conjugate pad, it is recognized by specific detection antibodies, and the immune complexes continue to move along until they are captured at the test line *via* a predeposited capturing antibody, which forms a double-antibody sandwich structure. The unreacted antibody is finally detected by a species-specific antibody at the control line, and unreacted reactants are absorbed by the absorbent pad. The aggregated latex beads on T line are dependent on the presence and concentration of PEDV; therefore, their concentration can be measured by a colorimetric assay. In the absence of PEDV, a sandwich-type immune complex cannot be formed, and no LBs aggregate at T line. Therefore, the presence of a red band caused by LB aggregation at T line could be used to detect the presence and concentration of PEDV (Fig. 1).

### Characterization of the LBs and LBs-mAbs conjugates

**Fig. 2:**
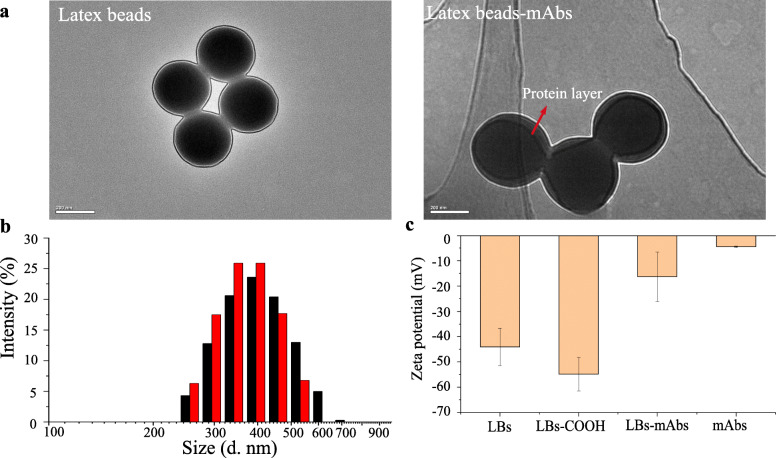
Characterization of the LBs-mAbs. **a** TEM images of LBs and LBs-mAb. **b** Hydrodynamic diameter distribution of LBs and LBs-mAbs. **c** Zeta potential distribution of LBs, activated LBs-COOH, LBs-mAbs and mAbs

The morphology and size of LB and prepared LB-mAb conjugates were characterized using transmission electron microscopy (TEM) and a Malvern laser particle size analyzer. As shown in Fig. 2a, the LBs are spherical particles with a uniform diameter of approximately 300 nm, whereas the surface of the LB-anti-PEDV-mAbs clearly shows an outer protein layer. The dynamic light scattering (DLS) results revealed that the average diameter of the LB-anti-PEDV-mAbs was 408.4 ± 92.15 nm, which was approximately 30 nm larger than that of the unbound LBs (375.8 ± 72.79 nm) (Fig. 2b). In addition, as shown by the zeta potential analysis, the negative charge was increased after activation of the carboxyl group on the surface of nanospheres. Surface charge of the activated latex beads was significantly reduced owing to antibody conjugation to the carboxylate-modified LBs (Fig. 2c).

Subsequently, to determine the mass of antibodies conjugated with each LB, supernatant from the LB-mAb reaction system were analyzed. BCA assay results showed that the concentration of protein in the supernatant from the LBs was 2.55 ± 1.29 μg, and the labeling efficiency of antibodies on the LBs was at least 91.46%, indicating that most of antibody proteins were conjugated to the LBs (Table S1). From the above analyses, we concluded that the LB-anti-PEDV-mAbs were successfully prepared.

### Optimization of the LFIA

It is known that the most challenging and complicated process during the development of LFIAs is the tuning of the different parameters and components to achieve a minimum amount or concentration that could be detected. Optimization of the developed LFIA relied on an iterative approach using trial and error (Hsieh et al. 2017; Parolo et al. 2020). The key factors in the LFIA system, including interpretation time, the amount of detecting antibody conjugated to the nanoparticle, and the captured antibody working concentration at T line, were systematically investigated to obtain the optimal performance. The optimal criteria for these parameters were determined by the test line gray value, which had to satisfy the demand for high sensitivity and specificity. All optimized processes were performed at room temperature to simulate the actual application.

First, to demonstrate the suitability of anti-PEDV paired antibodies for developing this LFIA system, a proof-of-concept assay experiment was performed using a short list of the most promising combinations, which confirmed anti-PEDV-A as the optimal detecting antibody and anti-PEDV-B as the optimal capturing antibody (Fig. S1). After screening the promising combinations, a range of interpretation times for the LBs-LFIA was tested (e.g., 5 to 30 min). As shown in Fig. S2, as the immunoreaction time was extended, the gray value of all reaction bands increased gradually until a reaction time of longer than 15 min, at which point there was a plateau. Thus, the interpretation time for PEDV detection was set at 15 min for subsequent experiments.

The amount of detecting antibodies conjugated to the LBs was critical to performance of the LFIA. The estimate of antibody concentration was based on the observation that the red bands on T line and C line became brighter with an increase in protein concentration (Fig. S3a). However, given the need to balance satisfactory performance and cost-effectiveness, 30 μg of anti-PEDV-mAbs was selected as the optimal mass (Fig. S3c).

In addition, the capturing antibodies immobilized on the test line have a significant influence on the analytical performance. Here, high and low concentrations of PEDV and a negative control test were used to optimize the concentrations of the capturing antibodies on T line. As shown in Fig. S3b, the color intensity of the red band on T line for the positive sample spiked as PEDV gradually increased with an increase in the NC membrane-immobilized capturing antibodies of the anti-PEDV-mAbs. However, the excess capturing mAbs on T line resulted in a lower color intensity for the positive samples (Fig. S3d). Thus, the optimal concentration of the capturing mAbs on T line was 0.75 mg/mL of anti-PEDV-mAbs (Table 1).

**Table.1:** Optimization of the LBs-LFIA condition

Experimental parameters	Condition	Optimal value
Interpretation time (min)	5, 10, 15, 20, 25, 30	15
The amount of mAbs labeled with LBs (μg)	10, 20, 30, 40, 50	30
Concentration of c-mAb on T line (mg/mL)	0.25, 0.5, 0.75, 1.0, 1.5	0.75

### Optimization of sample application for the LBs-LFIA

After optimizing the parameters of the LBs-LFIA, we investigated the application of swine fecal sample. Many factors influence antigen-antibody reaction; one important factor is pH. In this study, pH of a total of 70 PEDV-infected fecal samples and 38 healthy swine fecal samples were measured. pH values ranged from 5.5 to 7.5, and most of samples were acidic. The rationale of the buffer optimization was to minimize any nonspecific binding between reporter particles and the assay target without compromising the signal intensity. Therefore, modification of pH would be a good way to fine-tune the conformation of antigen-antibody reaction. Optimization of the running buffer revealed that pH 8.0 was suitable for the detection of swine feces and neutralized the substance to achieve the optimal antigen-antibody reaction (Fig. S4).

**Fig. 3:**
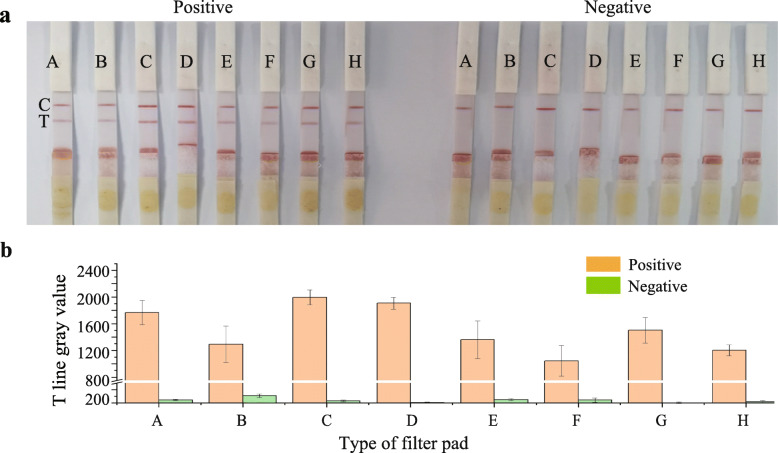
Optimization of filter pads added to the LBs-LFIA. **a** Images of the positive and negative sample results using different filter pads. **b** Gray value analysis of T line

Furthermore, considering the complex matrix of the swine fecal sample, the sample pad did not provide sufficient particle flow, and a blockage occurred between the conjugate pad and the sample pad, which induced the uncontrolled release of fecal sample liquid. Therefore, the effect of various filter pad materials were tested by using a swine fecal sample diluent to improve the selectivity (Table S2). The results demonstrated that filter pad V7 displayed the best performance, and the color intensity of filter pad decreased effectively relative to the LFIA without this additional pad (Fig. 3). Therefore, filter pad V7 was selected as the most suitable material for this assay, which reduced background variation and improved PEDV virion detection in feces on the LBs-LFIA.

### Investigation of LFIA performance

**Fig. 4:**
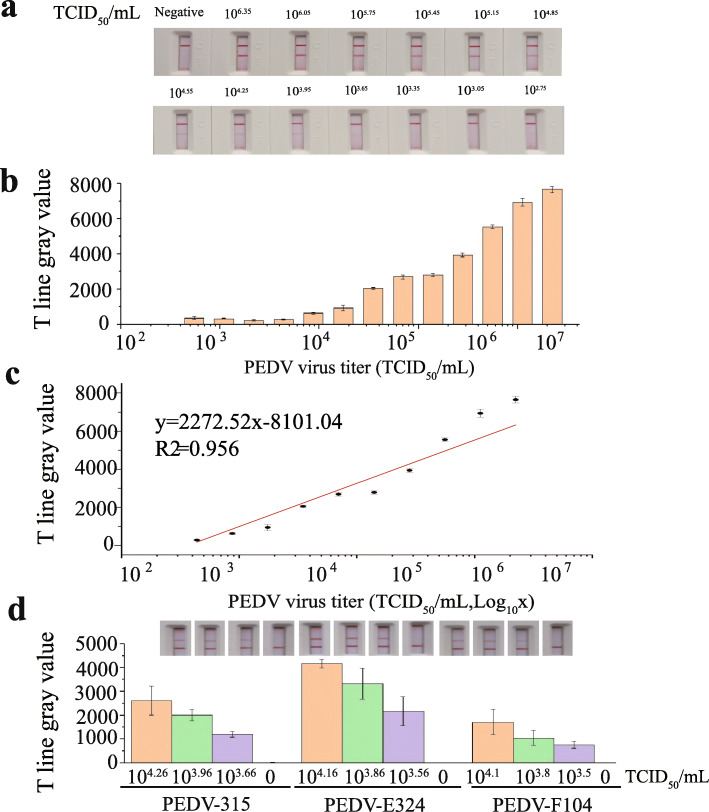
Analytical sensitivity of LBs-LFIA for PEDV detection **a** Photograph of visual interpretation **b** The dependence of the gray value of T line of LBs-LFIA on the concentration of PEDV **c** A calibration curve drawn of T line gray values in the range of 10^3.65^ to 10^6.35^ TCID_50_/mL **d** The LOD verification test for LBs-LFIA. PEDV, porcine epidemic diarrhea virus. Error bars represent the standard deviation of three replicate experiments

Under the optimized conditions, the analytical sensitivity of PEDV was evaluated with a range of viral titers. The viral titer was measured by the endpoint dilution assay. The stock solution of PEDV virus titer was 10^6.65^ TCID_50_/mL (80 μg/mL). As shown in Fig. 4a, 100 μL of sample solutions containing different concentrations of PEDV (from 10^2.75^–10^6.35^ TCID_50_/mL, 9.77–40,000 ng/mL) and the negative control were added to the sample pad hole. With an increase in PEDV concentration, T line on the LBs-LFIA appeared as a clear red band. The color intensity of T line, calculated from the gray value, was extracted from the image to realize semiquantitative analysis. The gray value of T line exhibited a linear relationship with the PEDV virus titer in the range of 10^3.65^ to 10^6.35^ TCID_50_/mL (Fig. 4c). The limit of detection (LOD) was calculated to be 10^3.60^ TCID_50_/mL, as defined by the mean gray value on T line of the blank control plus three standard deviations (formula: y_blank_ + [3× SD_blank_]). The color of red band on T line was completely absent when testing 10^3.95^ TCID_50_/mL of PEDV (Fig. 4a). Moreover, to verify whether the LOD was applied to different PEDV samples with temporal and regional differences and to assess the detection rates near the estimated LOD, three PEDV strains were diluted to approximately 10^4^ TCID_50_/mL (Fig. S5). As illustrated in Fig. 4d, the detection rate for this low concentration of PEDV was 100%, and this was therefore considered the LOD.

**Fig. 5:**
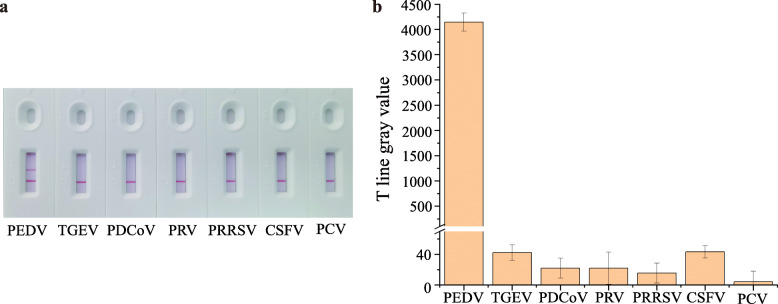
Analytical specificity. **a** Photograph of common swine virus tested by LBs-LFIA. **b** Gray values of T line on the LBs-LFIAs

To further evaluate the specificity of LBs-LFIA for PEDV recognition, several general swine disease viruses, such as TGEV, PDCoV, pseudorabies viruspseudorabies virus (PRV), porcine reproductive and respiratory syndrome virus (PRRSV), classical swine fever virus (CSFV) and porcine circovirus (PCV) were selected as controls. Images were captured in daylight using a smartphone camera, and the gray values of T line color intensity were analyzed using ImageJ software (Fig. 5b). As shown in Fig. 5a, the presence of PEDV made T line appear as a remarkable red band in daylight, whereas there was no obvious color change for the controls. Meanwhile, analysis of the gray value confirmed this result, indicating the high specificity of LBs-LFIA. The accuracy of LBs-LFIA at three virus concentrations was 0.182, 1.994, and 22.879, which met the requirement to be within 15% of the nominal value for the detection range (Table 2). The recovery rate of the added PEDV was between 91.00% and 114.40%, showing the excellent accuracy of the LBs-LFIA.

**Table.2:** Recovery of LBs-LFIA in PEDV-spiked swine fecal samples

Added (μg/mL)	Found (μg/mL)	Recovery^a^ (%)	StD^b^	CV^c^ (%)
20	22.879	114.40	2.262	9.89
2	1.994	99.70	0.202	10.1
0.2	0.182	91.00	0.022	11.0

^a^Recovery (%) = (Detected concentration/spiked concentration) × 100%

^b^StD, Standard deviation (*n* = 5)

^c^CV, Coefficient variation = (Mean/StD) × 100%

### Stability of the LBs-LFIA

**Fig. 6:**
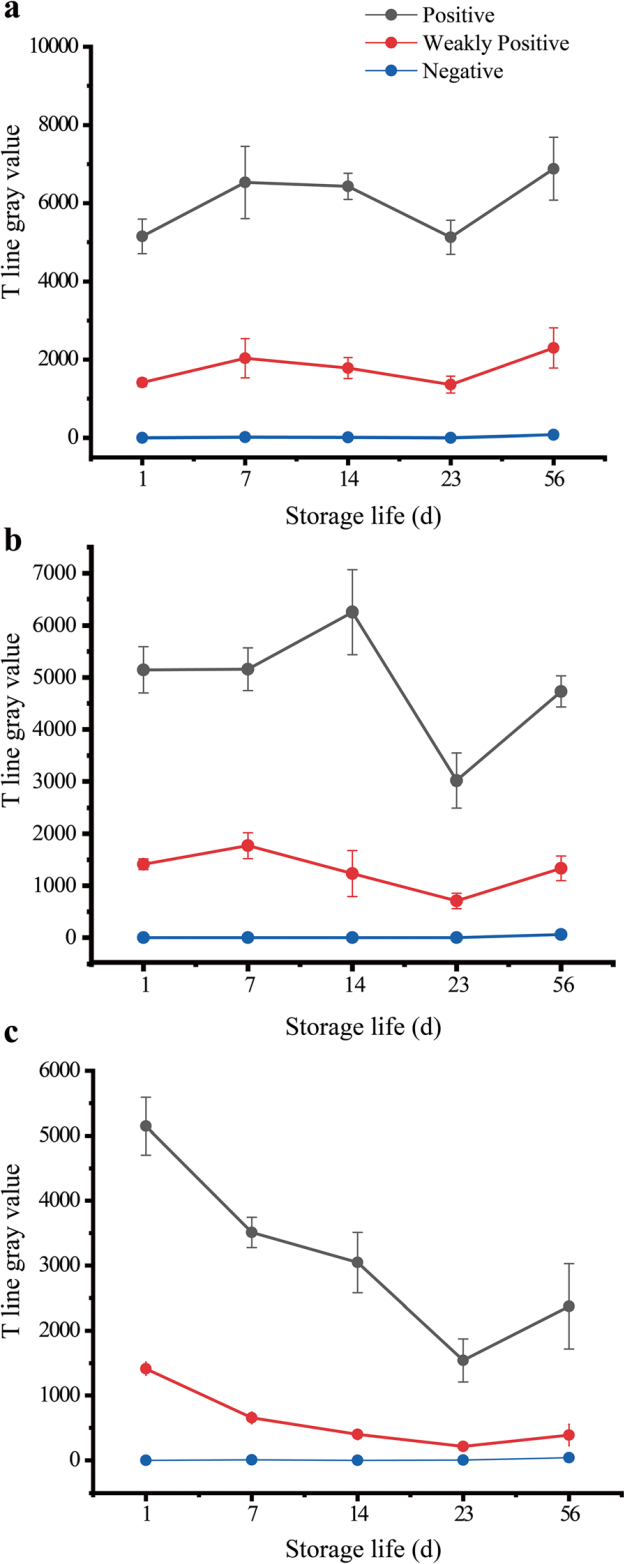
Gray values of T line for the detection of high and low PEDV concentrations and the negative control over time. **a** Room temperature **b** 37 °C **c** 50 °C

In the present study, stability of the LBs-LFIA to high and PEDV oncentrations and the negative control was tested after the device was stored for 7, 14, 23 and 56 consecutive days. As shown in Fig. 6a, at RT, the LBs-LFIA showed a stable PEDV detection performance for 56 days. Meanwhile, the color intensity on T line of the LBs-LFIA decreased after 56 days of storage at 37 °C and 23 days of storage at 50 °C; however, its qualitative performance remained relative to the initial results (Fig. 6). Thus, we concluded that the LBs-LFIA could be stored at RT for at least 56 days, at 37 °C for at least 56 days, and at 50 °C for at least 23 days.

### Qualitative detection of PEDV in clinical fecal samples

**Fig. 7:**
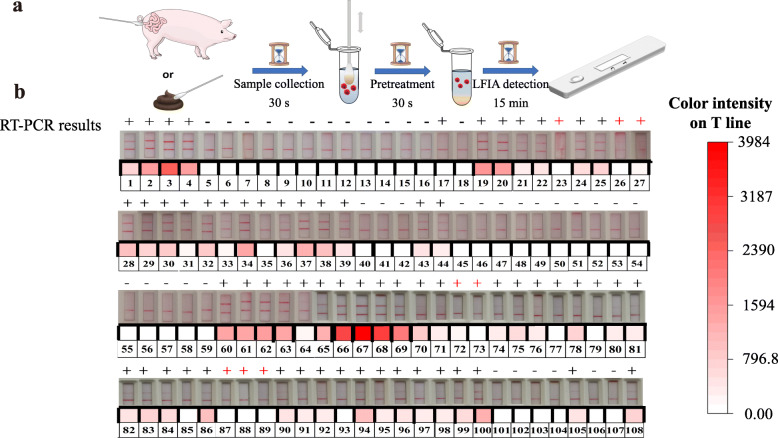
Detection of PEDV in clinical swine fecal samples. **a** Workflow and turnaround time for clinical PEDV detection with LBs-LFIA. **b** Swine fecal sample detection results. Each sample was confirmed by RT-PCR in advance

To explore the potential use of this LB-LFIA for PEDV detection, we evaluated the clinical feasibility of this platform for the selection of swine fecal samples confirmed by RT-PCR to have 70 PEDV-positive samples and 38 PEDV-negative samples. As shown in Fig. 7a, the turnaround time for the entire workflow, including receiving a result, was approximately 16.5 min: 30 s sample collection, 30 s sample pretreatment, and 15 min processing time on the LBs-LFIA strip.

Of the 108 swine fecal samples, all negative RT-PCR results were negative in the LBs-LFIA. Eight swine fecal samples that were positive by RT-PCR were negative in the LBs-LFIA, and the remaining samples were positive by both RT-PCR and in the LBs-LFIA (Fig. 7b, Table 3). Cohen’s kappa (κ) is a frequently used metric of the reliability of agreement between categorical variables. As shown in Table S3, the results from the LBs-LFIA were in almost complete agreement with the RT-PCR results (κ = 0.845, 95% confidence interval [CI]: 0.793–0.897, *P* < 0.001). In conclusion, in the clinical sample analysis, the developed LBs-LFIA for POC diagnosis of clinical swine fecal samples showed great potential for monitoring PEDV infection in the field.

**Table.3:** Comparison of the analysis results between LBs-LFIA and RT-PCR

Analytical methods		RT-PCR		Total
		Positive	Negative	
LBs-LFIA	Positive	62	0	62
	Negative	8	38	46
	Total	70	38	108
	Coincidence rate (%)	88.57	100	92.59

## Discussion

Effective diagnosis of animal pathogens requires rapid, sensitive and specific techniques that can be used for routine diagnosis in the laboratory and in the field. In recent years, LFIA has been widely used in clinic because of its rapid detection, easy operation, and cost-effectiveness. In our study, a paper-based lateral flow immunoassay for PEDV detection in swine fecal samples was developed using color-rich latex beads as the label. The analytical performance showed high sensitivity (LOD = 10^3.60^TCID_50_/mL), no cross-reactions with other pathogens, and qualified stability.

In our previous clinical test, we found that solid impurities in swine feces reduced the accuracy of the immunoassay. To solve this problem, we added a filter pad between the sample pad and the conjugate pad to improve the accuracy of the LB-LFIA for swine fecal analysis. Compared with previously prepared LFIAs, in this study, the performance of our test strips was improved by adding a filter pad between the conjugate pad and the sample pad. The potential reason for this improvement is that the filter pad design can efficiently eliminate any nonspecific reaction by preventing nonspecific retention of fecal particulate impurities in the test line area. Moreover, this additional pad design can separate target antigens to improve antigen-antibody interactions in the conjugate pad. In particular, in clinical fecal sample detection, the additional filter pad design showed a positive effect on the LFIA platform test.

Compared with real-time RT-PCR, the sensitivity and specificity of this method were 88.57% and 100%, respectively, and a total coincidence rate of 92.59% was maintained with the observed results in experimentally infected piglets. In addition, the test only needs naked eye observation to interpret the results without the need for professional personnel and sophisticated instruments. On the one hand, this is beneficial for PEDV diagnosis in remote areas or pig farms; on the other hand, different individuals with subjective judgments could lead to different interpretations of weak positivity. To obtain a more accurate analysis method, we may design a handheld reader based on mobile phones to read the information of the LFIA strip in the future to make the results more precise, objective and sensitive.

In conclusion, we successfully developed an LBs-LFIA method for rapid and accurate detection of PEDV, providing a flexible application in LFIA platform development. We also believe this filter pad design has the potential to overcome other clinical detection problems.

## Conclusions

We have successfully developed an easy-to-use LB-LFIA for the rapid detection of PEDV in swine fecal samples. The LOD of this method was 10^3.60^ TCID_50_/mL, and there was no cross-reactivity with other related swine viruses. The results of the clinical sample tests indicated that the LBs-LFIA had stable and accurate analytical performance for PEDV diagnosis, which is appealing and promising for on-site analysis and user-driven on-site testing in pig farms.

## Methods

### Reagents and apparatus

Anti-PEDV monoclonal antibodies A and B, PEDV strain CHYJ130330, TGEV, PDCoV, PRV, PRRSV, CSFV and PCV were obtained from Guangdong Haid Institute of Animal Husbandry & Veterinary (Guangdong, China). LBs, N-(3-dimethyaminopropy)-N′-ethylcarbodiimide hydrochloride (EDC), and N-hydroxysulfosuccinimide (sulfo-NHS) were purchased from Thermo Fisher Scientific (Fremont, USA). 2-(N-morpholino) ethanesulfonic acid (MES) and bovine serum albumin (BSA) were purchased from Sigma-Aldrich (Shanghai, China). Goat anti-mouse IgG was purchased from Artron (Shandong, China). Nitrocellulose (NC) membranes, sample pads, filter pads, conjugation pads, plastic backing, and absorbent pads were purchased from Shanghai JieNing Biotech Co., Ltd. (Shanghai, China). Ultrapure water was produced by a Milli-Q Ultra Pure System (Millipore, USA) and used throughout the study. All chemicals were of analytical grade or higher.

A centrifuge (HITACHI, Japan), XW-80 vortex mixer (Shanghai, China), ultrasonic homogenizer (Ningbo, China), programmable strip cutting machine HGS201 (AUTOXUN, China), XYZ3060 platform (Bio-Dot Scientific Equipment, USA), and an electric forced air convection drying oven (TAISITE Instrument, China) were used to produce the LFIA. A transmission electron microscope (Philips, Holland) and a particle size analyzer (Malvern, UK) were used to characterize the LBs.

### Preparation of LBs-mAbs conjugates

The color-rich dyed latex beads were conjugated with anti-PEDV-monoclonal antibodies by heterobifunctional cross-linking using EDC and sulfo-NHS, which preactivated the carboxyl groups on the particles to allow easy binding to the free amino group of the antibodies to form stable amide bonds. Before the binding reaction was started, 1% (*w*/*v*) EDC and 1% (*w*/*v*) sulfo-NHS solution were successively added to 0.2% (*w*/*v*) LB solution in a 1 to 5 ratio. After reacting on a rotary mixer for 30 min at room temperature, the activated LBs were separated by centrifugation at 20,000 *g* for 30 min at 4 °C and resuspended in 0.1 M MES buffer by sonication to assist in the redispersion of the clumped particles. Subsequently, 20 μg of anti-PEDV monoclonal antibodies, as the detecting mAbs, were added to the activated LB solution, and the reaction tube was placed on a rotary mixer for 90 min. Then, 10 μL of 10% (*w*/*v*) BSA solution was added to 1 mL of binding reaction system to block nonspecific binding, and the solution was rotated gently for 60 min at room temperature. Finally, the solution containing antibody-conjugated LBs was separated by centrifugation at 20,000 *g* for 30 min at 4 °C. The precipitate was resuspended in a half volume of glycine-NaOH buffer (0.1 M, pH 8.5) containing 8% (*w*/*v*) sucrose, 2% (*w*/*v*) trehalose, 1% (*w*/*v*) BSA, 0.5% (*w*/*v*) sodium casein, and 0.02% (*w*/*v*) sodium azide, and the particles were dispersed in solution by ultrasonication.

### Fabrication of the LBs-LFIA strips

To prepare the LFIA strips, the pretreated sample pad, filtration pad, conjugate pad, NC membrane, and absorbent pad were deposited onto a polyvinyl chloride card with an overlap of 1–2 mm in length. The sample pad was soaked with phosphate buffer (0.2 M, pH 8.0) containing 2.5% (*w*/*v*) sucrose, 1% (*v*/*v*) Tween-20, and 0.05% (*v*/*v*) ProClinTM300 and dried at room temperature for 10 h. A piece of the NC membrane (width, 25 mm; length, 300 mm) was used to immobilize 0.75 mg/mL anti-PEDV-capturing monoclonal antibodies and 3 mg/mL goat anti-mouse IgG polyclonal antibodies in distinct zones with a volume of 0.5 μL/cm using a dispenser and then dried at 37 °C overnight. Each LB-LFIA was assembled by superposition of the different pads and then was cut into 3.8 mm wide single-use strips that were housed in a cassette and stored in bags containing desiccant until use.

### Assay procedure for the LBs-LFIA

The following assay procedure was used for the LBs-LFIA: 100 μL of running buffer was mixed with the 1/2 swab (appropriate 0.5 g) sample of pig feces, and then the supernatant was transferred to the sample pad of the LFIA. Upon addition of a liquid sample, the particles on the conjugate pad were rehydrated, and the antigen (if present) and the sample migrate *via* capillary action through the test line (T line) and control line (C line) on the NC membrane. After reacting for 15 min, the gray value of the red band on T line that reflects the virus concentration was recorded by a smartphone camera and analyzed by ImageJ software.

### Optimization of key parameters of the LBs-LFIA and practical application

The interpretation time of the LBs-LFIA, the amount of mAbs labeled with LBs, the concentration of capturing antibodies on T line, and the pH of the running buffer were considered critical parameters governing the analytical performance of the developed LBs-LFIA. Following optimization, we investigated the application of the LBs-LFIA for the analysis of swine fecal samples and addressed the problems impairing the performance of the LBs-LFIA by optimizing the running buffer and adding the filter pad.

A single-factor analysis was applied to optimize the parameters in this study. The details of the condition optimization of the LFIA system are provided in Table 1.

### Evaluation of the analytical performance of the LBs-LFIA

To evaluate the analytical sensitivity of the LBs-LFIA, different known concentrations of PEDV were spiked in sample diluent mixed with appropriate PEDV-negative swine feces, and serial dilutions of the starting sample were prepared. After mixing the diluent, 100 μL of the supernatant mixture was added to the sample pad hole. Each concentration was analyzed in triplicate. The results were diagnosed by the naked eye, as the colorimetric change provided suitable visual analytical sensitivity. Images of T line captured with a smartphone camera were also processed to obtain the corresponding gray values to quantify the analytical performance of the LBs-LFIA. The standard curve was generated from a known amount of virus, and the gray values of T line were used to determine the analytical sensitivity. Moreover, after determining LOD of the LBs-LFIA, we further verified LOD with different PEDV strains. The specificity of the LBs-LFIA was determined by analysis of different common swine viruses prepared at the same concentration. The accuracy was determined by replicate analysis of LBs-LFIAs that contained known PEDV concentrations, with the measurement of five determinations for each analyte concentration.

### Stability of the LBs-LFIA

The stability of an LB-LFIA product is a critical factor in its suitability in research and development (R&D). In this study, to estimate the storage stability of LBs-LFIA, the same batch of LBs-LFIA was divided into three subgroups, which were stored separately at room temperature (RT, 25 °C), 37 °C and 50 °C. The LB-LFIAs were evaluated for the desired performance using actual storage condition tests and accelerated aging studies. Before beginning the shelf-life evaluation, the LB-LFIA strips were packed in aluminum foil bags with desiccant. Fifteen pouches were tested on the first day. Forty-five pouches were each maintained at 37 °C and 50 °C in a drying oven, and 45 pouches were stored at RT for 56 consecutive days. Pouches were taken from each of the storage conditions after 7, 14, 23 and 56 d, and the LB-LFIA strips were used to analyze a high concentration of PEDV, a low concentration of PEDV, and a negative control. Five replicates were performed. The qualitative judgment standard was based on the presence of a red band on T line.

### Clinical application of LBs-LFIA for swine fecal samples

To verify the feasibility and practicability, 108 swine fecal samples were analyzed for the presence of PEDV using the LBs-LFIA. The presence of PEDV in the swine fecal samples was first confirmed by RT-PCR, which is defined as the gold standard for PEDV detection. As illustrated in Fig. 7a, the swine feces test procedure comprised sample collection, sample pretreatment, and LB-LFIA analysis. Finally, the LB-LFIA detection results were compared with the RT-PCR results to validate the qualitative analysis of the swine fecal samples.

## Supplementary Information

**Additional file 1.**

## Data Availability

All data can be shared upon reasonable request.

## Abbreviations

PEDV: porcine epidemic diarrhea virus
LBs-LFIA: latex bead-based lateral flow immunoassay
LOD: limit of detection
CV: coefficient of variance
PED: porcine epidemic diarrhea
TGEV: transmissible gastroenteritis virus
PDCoV: porcine delta coronavirus
IFA: immunofluorescence assay
ELISA: enzyme-linked immunosorbent assay
qRT-PCR: quantitative real-time-reverse transcription-polymerase chain reaction
RT-LAMP: reverse transcription loop-mediated isothermal amplification
LFAs: lateral flow assays
LBs: latex beads
TEM: transmission electron microscopy
DLS: dynamic light scattering
SD: standard deviations
CI: confidence interval
PRV: pseudorabies virus
PRRSV: porcine reproductive and respiratory syndrome virus
CSFV: classical swine fever virus
PCV: porcine circovirus
EDC: N-(3-dimethyaminopropy)-N′-ethylcarbodiimide hydrochloride
sulfo-NHS: N-hdroxysulfosuccinimide
MES: 2-(N-morpholino) ethane sulfonic
BSA: bovine serum albumin
NC: nitrocellulose membrane
R&D: research and development
RT: room temperature
