# Supplementary material for: Development of an accurate lateral flow immunoassay for PEDV detection in swine fecal samples with a filter pad design
Source: Anim Dis. 2021 Nov 8;1(1):27. doi: 10.1186/s44149-021-00029-1 (PMC8572657; doi:10.1186/s44149-021-00029-1)
Supplement: Supplementary file 1 — Additional file 1. [file 44149_2021_29_MOESM1_ESM.zip › Supplementary Material1.docx]

Supplementary Material

**Development an accurate lateral flow immunoassay for PEDV detection in swine fecal samples with a filter pad design**

Siyi Zou ^a,1^, Lei Wu ^a,1^, Gan Li ^a,1^, Juan Wang^b^, Dongni Cao ^a^, Tao Xu ^a^, Aiqing Jia ^b,*^and Yong Tang ^a,^*

^a^Department of Bioengineering, Guangdong Province Engineering Research Center of Antibody drug and Immunoassay, Jinan University, Guangzhou, China

^b^Guangdong Haid Institute of animal Husbandry & Veterinary, China

*Corresponding author. Tel: +86 020 85223718. E-mail address: [tyjaq7926@163.com](mailto:tyjaq7926@163.com) (Y. Tang). Tel: +86 020 34800478. E-mail address: [59492815@qq.com (A](mailto:59492815@qq.com%20(A). Jia)

1 These authors contributed equally to this work.


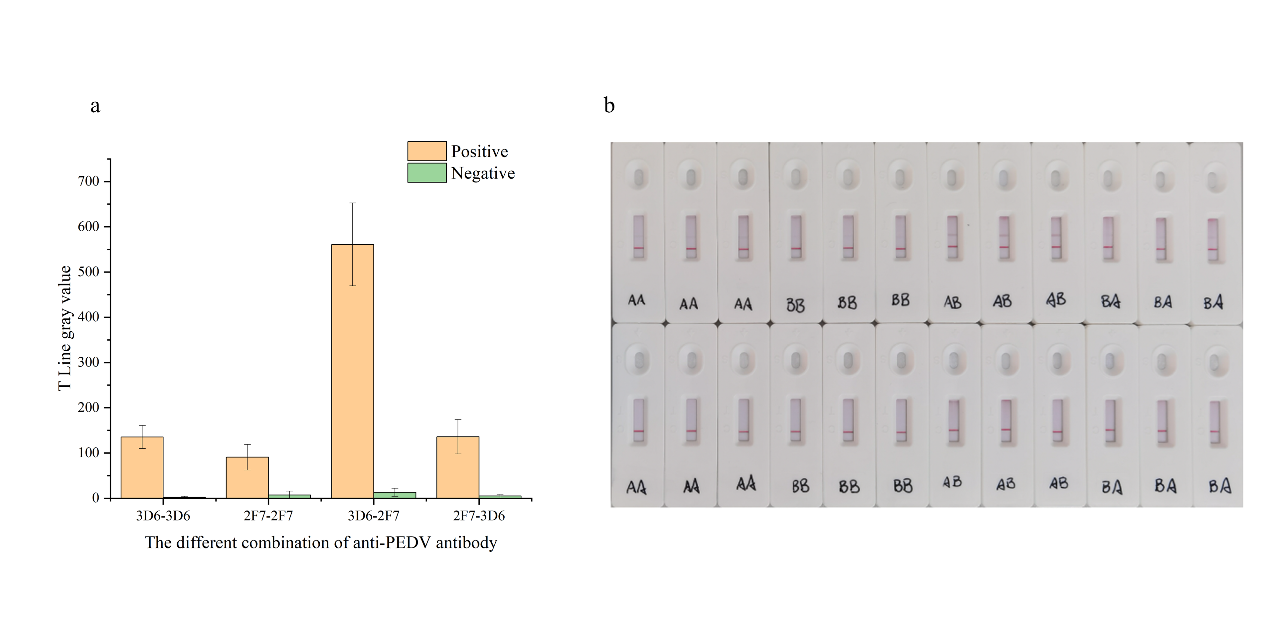


**Figure S1** Checkerboard test for paired antibodies. (a) Gray value analysis of T line (b) Photograph of results (A repressed anti-PEDV-A-mAbs, B repressed anti-PEDV-B-mAb)


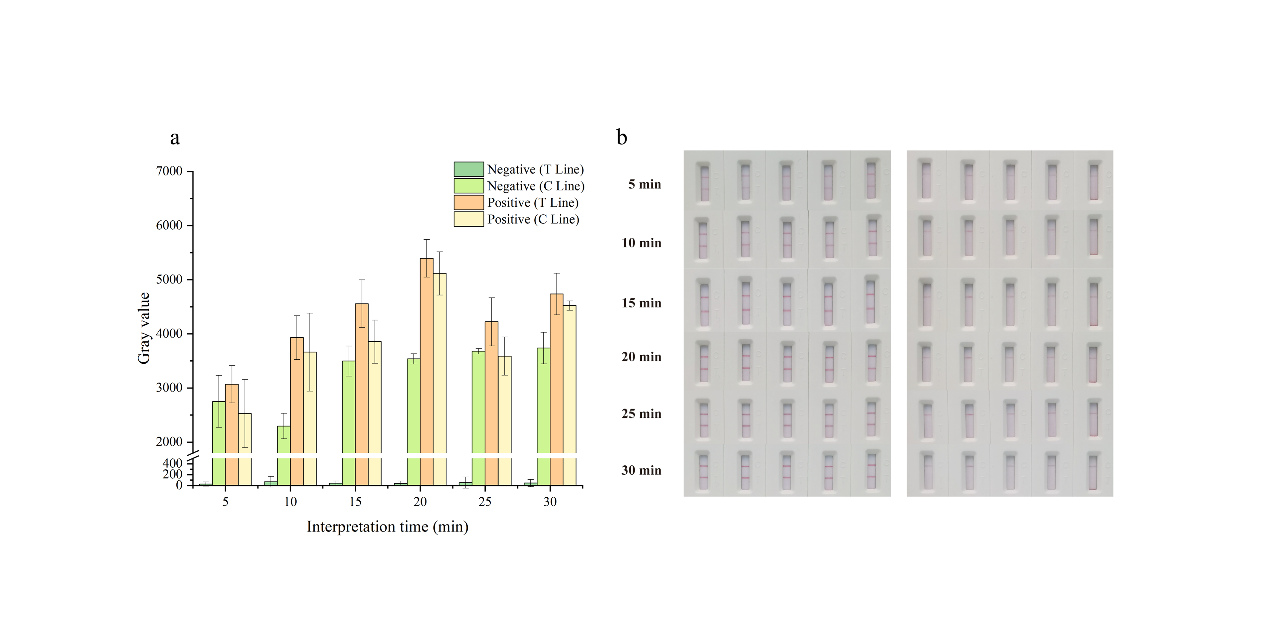


**Figure S2** Optimization of interpretation time (a) Relationship between time and gray value variation of both T line and C line (b) Photograph of interpretation time results


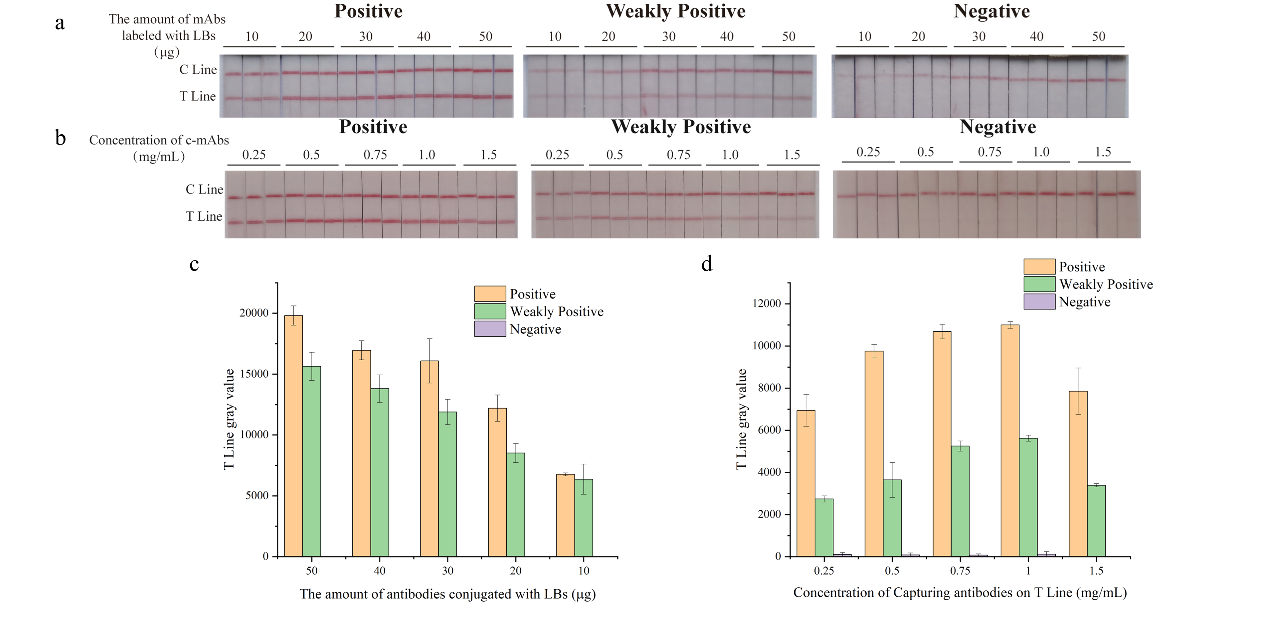


**Figure S3** Optimization of mAbs labeling amount and capture antibody concentration (a) Photograph of amount of mAbs labeled with LBs (b) Photograph of concentration of capturing mAbs (c) Result of amount of mAbs labeled with LBs (d) Result of concentration of capturing mAbs


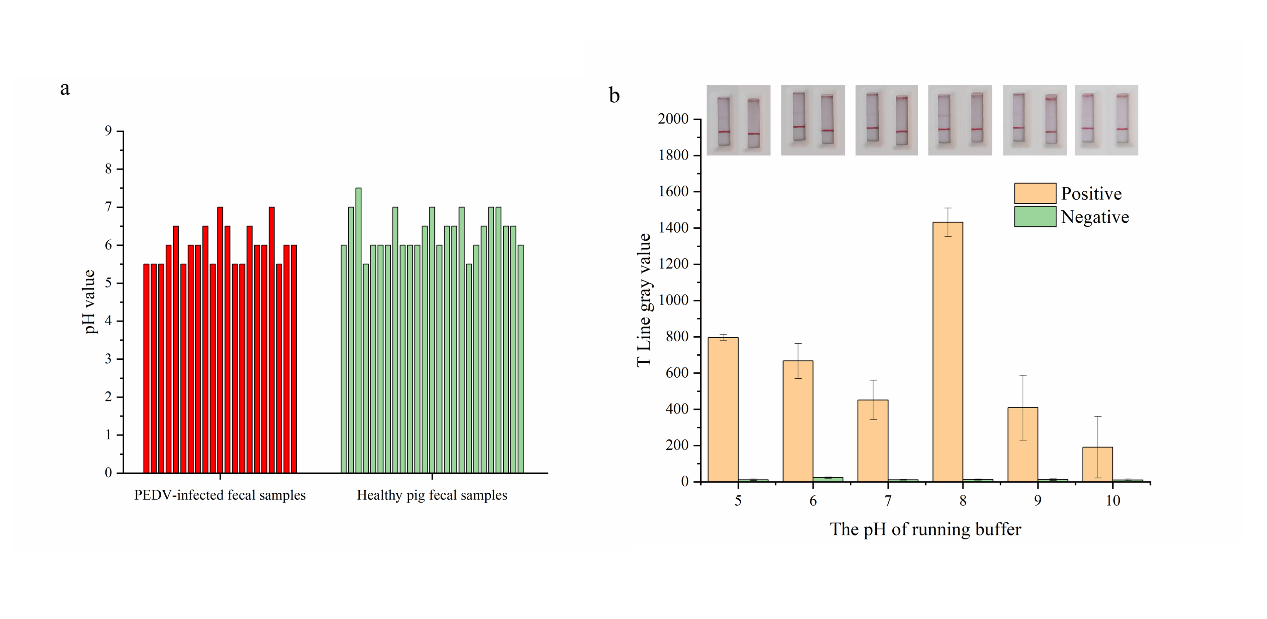


**Figure S4** (a) pH value of PEDV-infected and healthy fecal samples (b) Optimization of the pH of running buffer


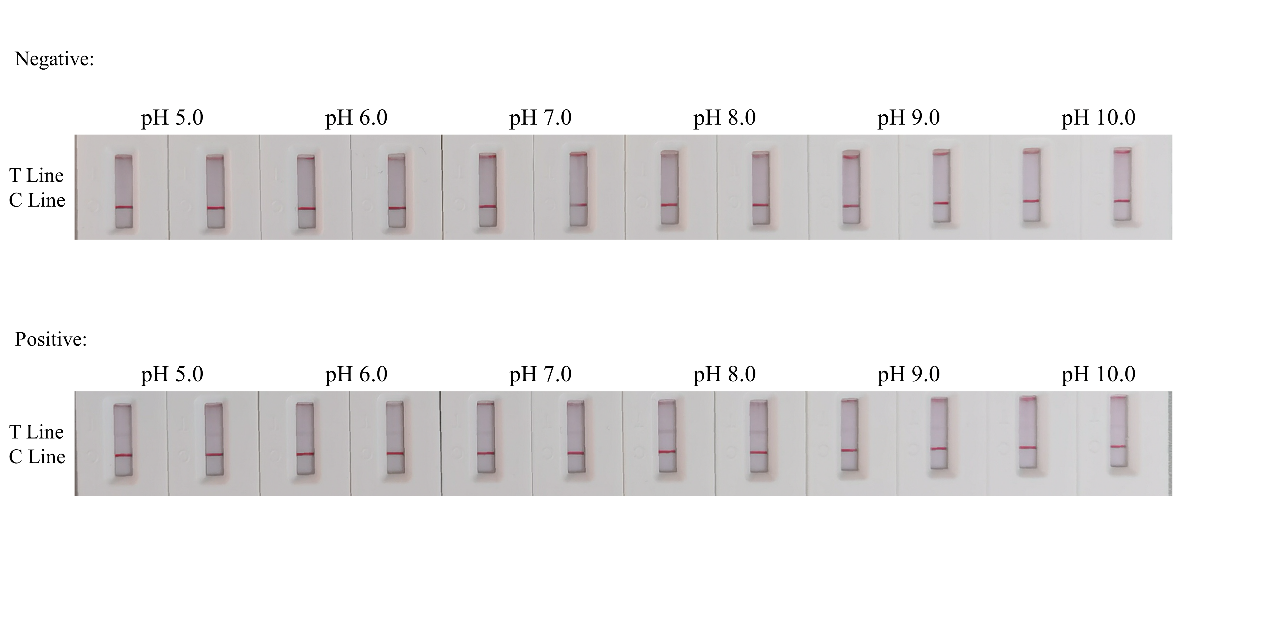


**Figure S5** Photograph of optimization of running buffer pH.


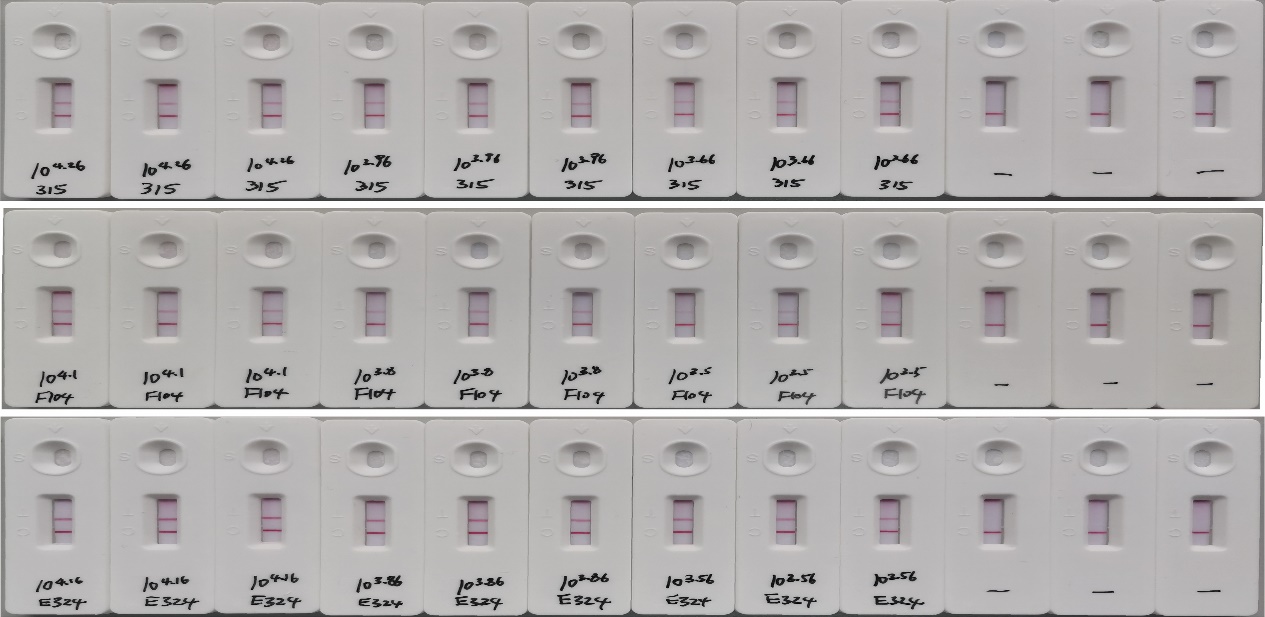


**Figure S6** Photograph of LOD verification experiments


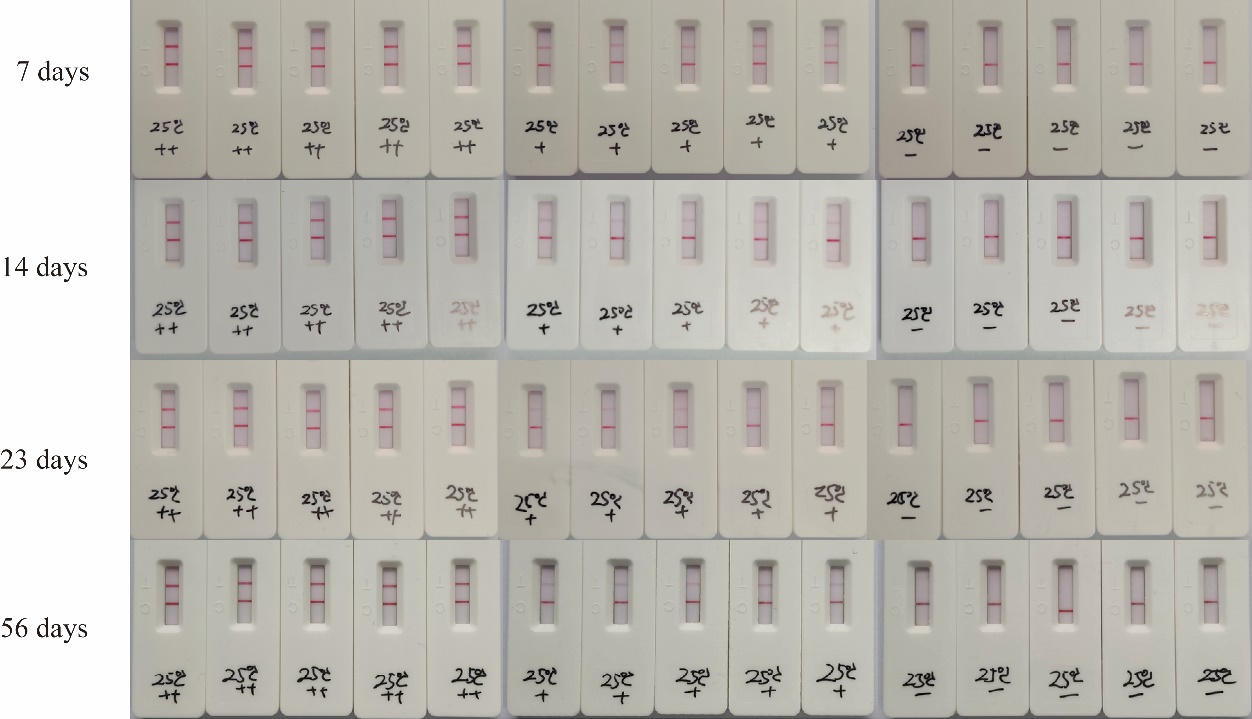


**Figure S7** Storage test of LBs-LFIA at room temperature for 56 days


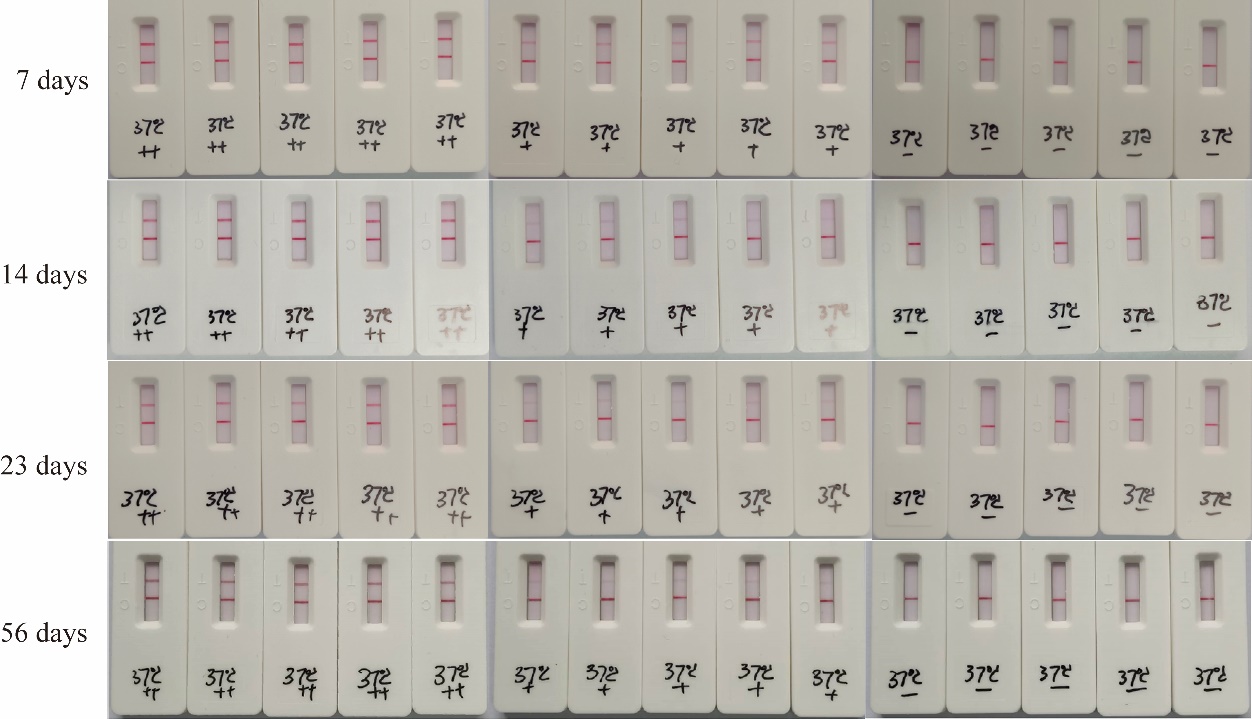


**Figure S8** Storage test of LBs-LFIA at 37°C for 56 days


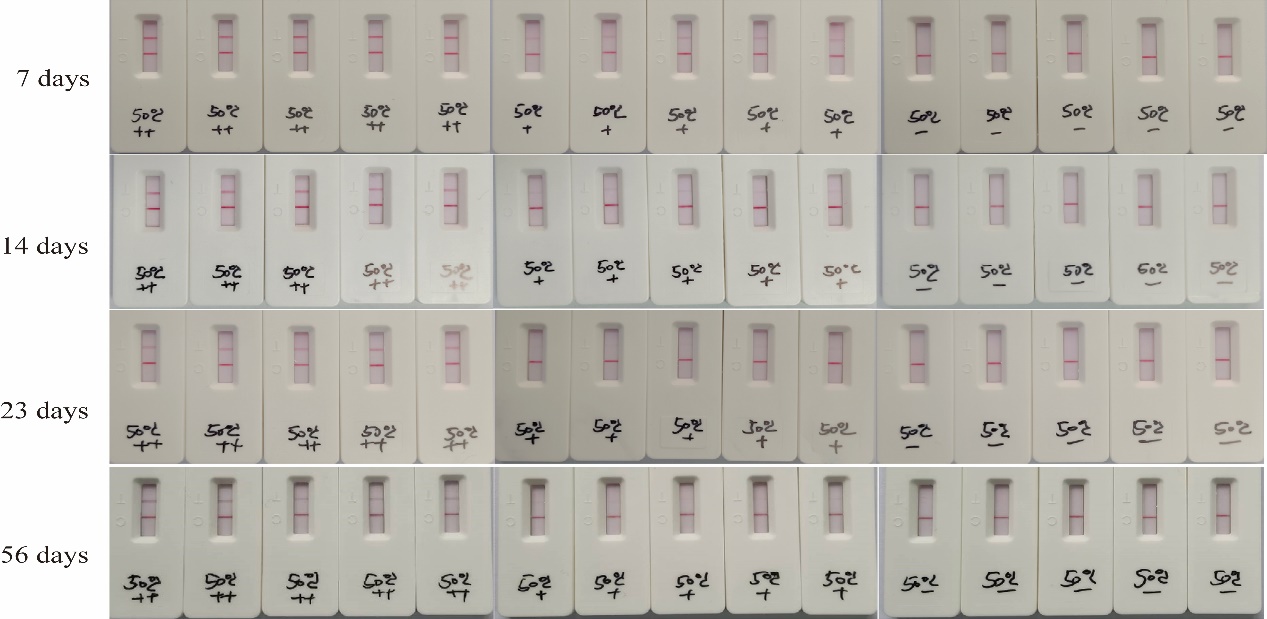


**Figure S9** Storage test of LBs-LFIA at 50°C for 56 days

**Table S1** Conjugated protein determined by BCA assay

| Added mAb proteins (μg) | Supernatant proteins on LBs (μg) | mAb proteins conjugated to LBs (μg) | Conjugated efficiency (%) |
| --- | --- | --- | --- |
| 29.83 | 3.46 | 26.37 | 88.41 |
|  | 1.64 | 28.19 | 94.51 |

**Table S2** Optimization of the type of filter pad

| No. | Filter pad composition | Type | Thickness (μm) |
| --- | --- | --- | --- |
| A | None | None | None |
| B | Mixed | Fusion 5 | 370 |
| C | Plasma Separation Membrane | V-7 | 316 |
| D |  | V-9 | 800 |
| E | Glass cellulose | GL-b04 | 750 |
| F |  | Ahlstrom 8964 | 410 |
| G | Polyester Fibers | Ahlstrom 6613 | 419.1 |
| H |  | Ahlstrom 6615 | 510 |

**Table S3** Coefficient of internal consistency

|  |  | Value | Asymp. Std. Error^a^ | Approx. T^b^ | Approx. Sig. |
| --- | --- | --- | --- | --- | --- |
| Measure of Agreement | Kappa | .845 | .052 | 8.889 | .000 |
| N of Valid Cases |  | 108 |  |  |  |

Note:

a. Not assuming the null hypothesis

b. Using the asymptotic standard error assuming the null hypothesis
