# Supplementary figures and images for: Development of an accurate lateral flow immunoassay for PEDV detection in swine fecal samples with a filter pad design
Source: Anim Dis. 2021 Nov 8;1(1):27. doi: 10.1186/s44149-021-00029-1 (PMC8572657; doi:10.1186/s44149-021-00029-1)

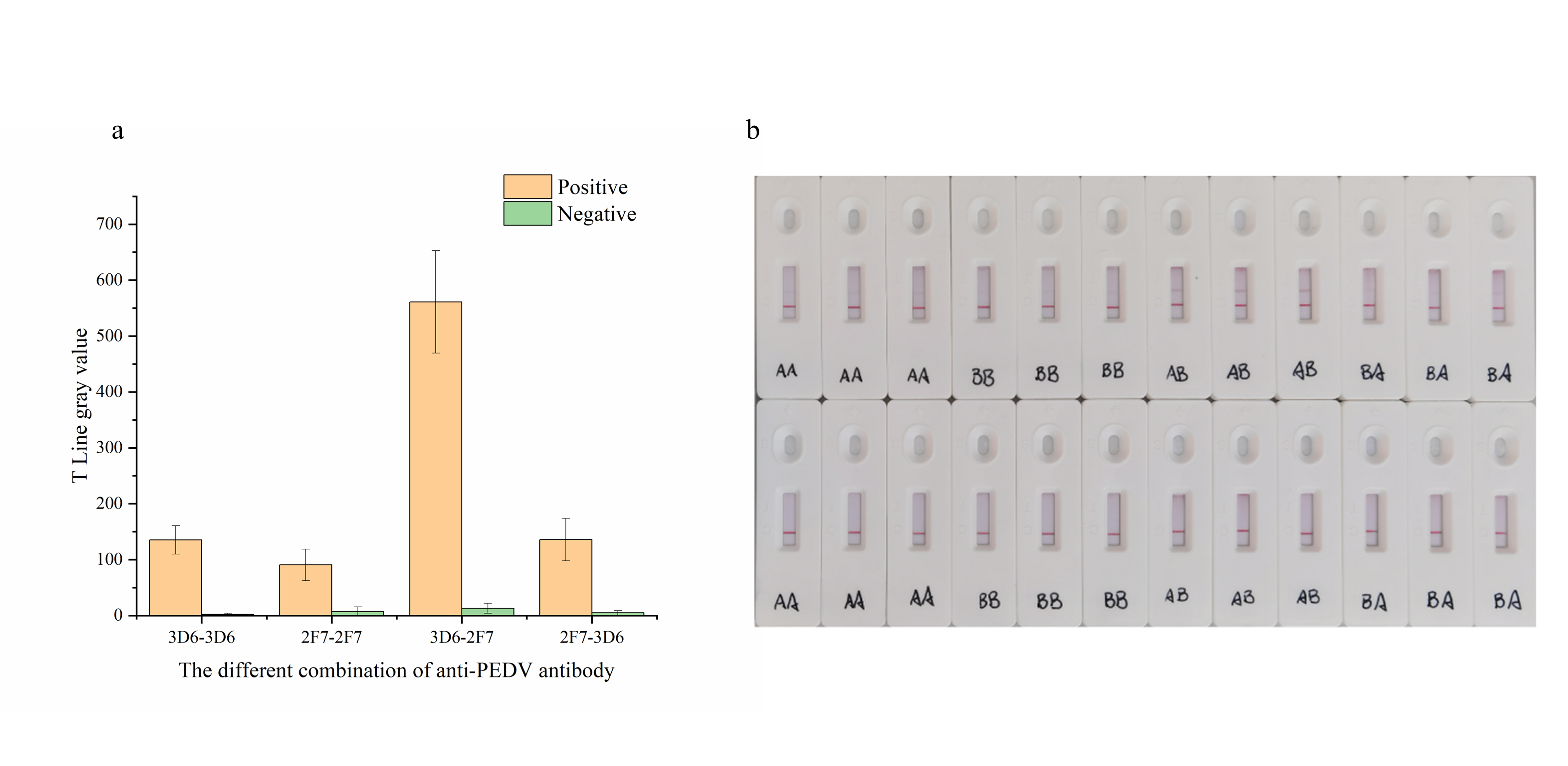

Supplement: Supplementary file 1 — Additional file 1. [file 44149_2021_29_MOESM1_ESM.zip › Fig S1.tif.tif]

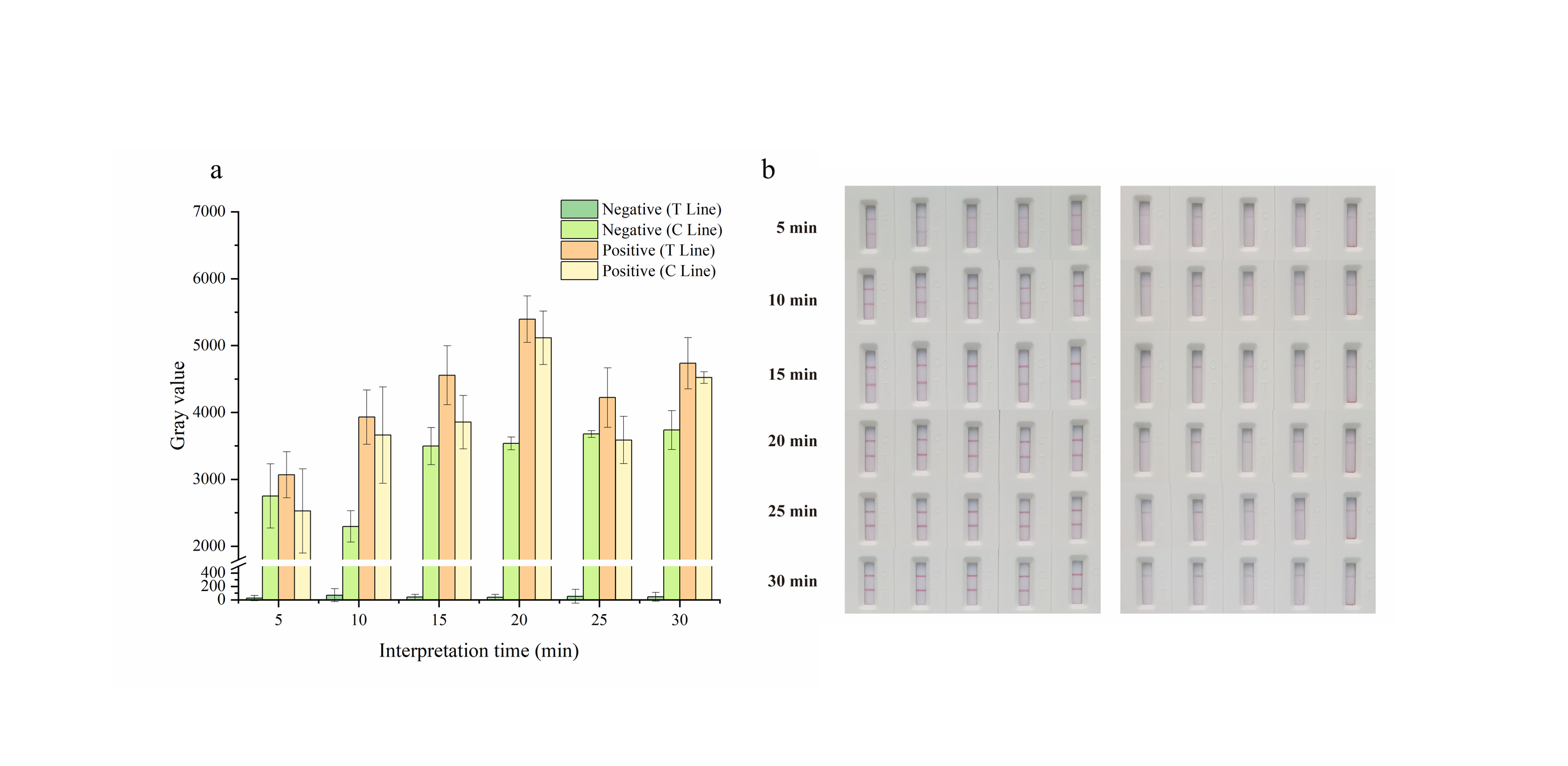

Supplement: Supplementary file 1 — Additional file 1. [file 44149_2021_29_MOESM1_ESM.zip › Fig S2.tif.tif]

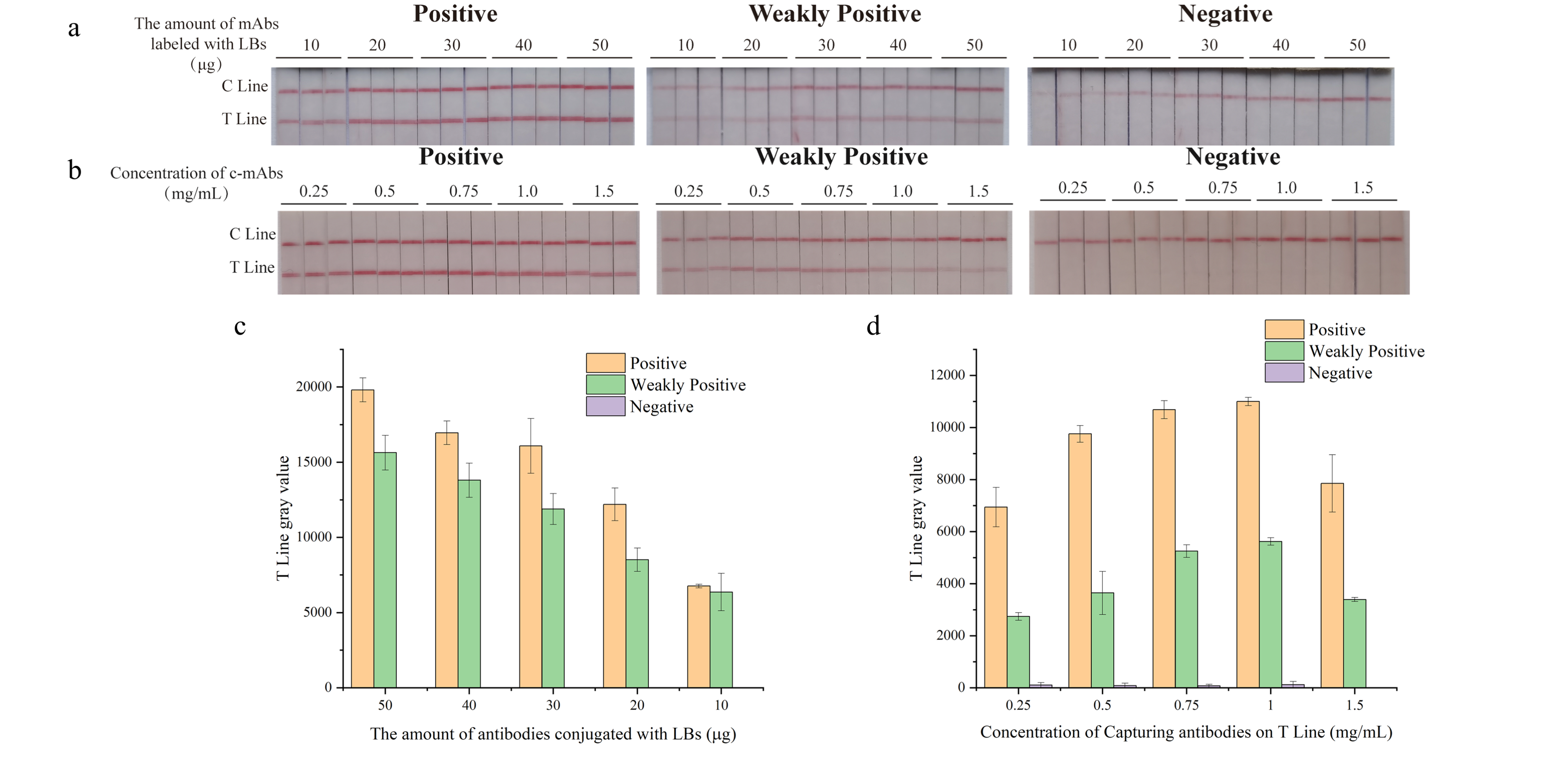

Supplement: Supplementary file 1 — Additional file 1. [file 44149_2021_29_MOESM1_ESM.zip › Fig S3.tif.tif]

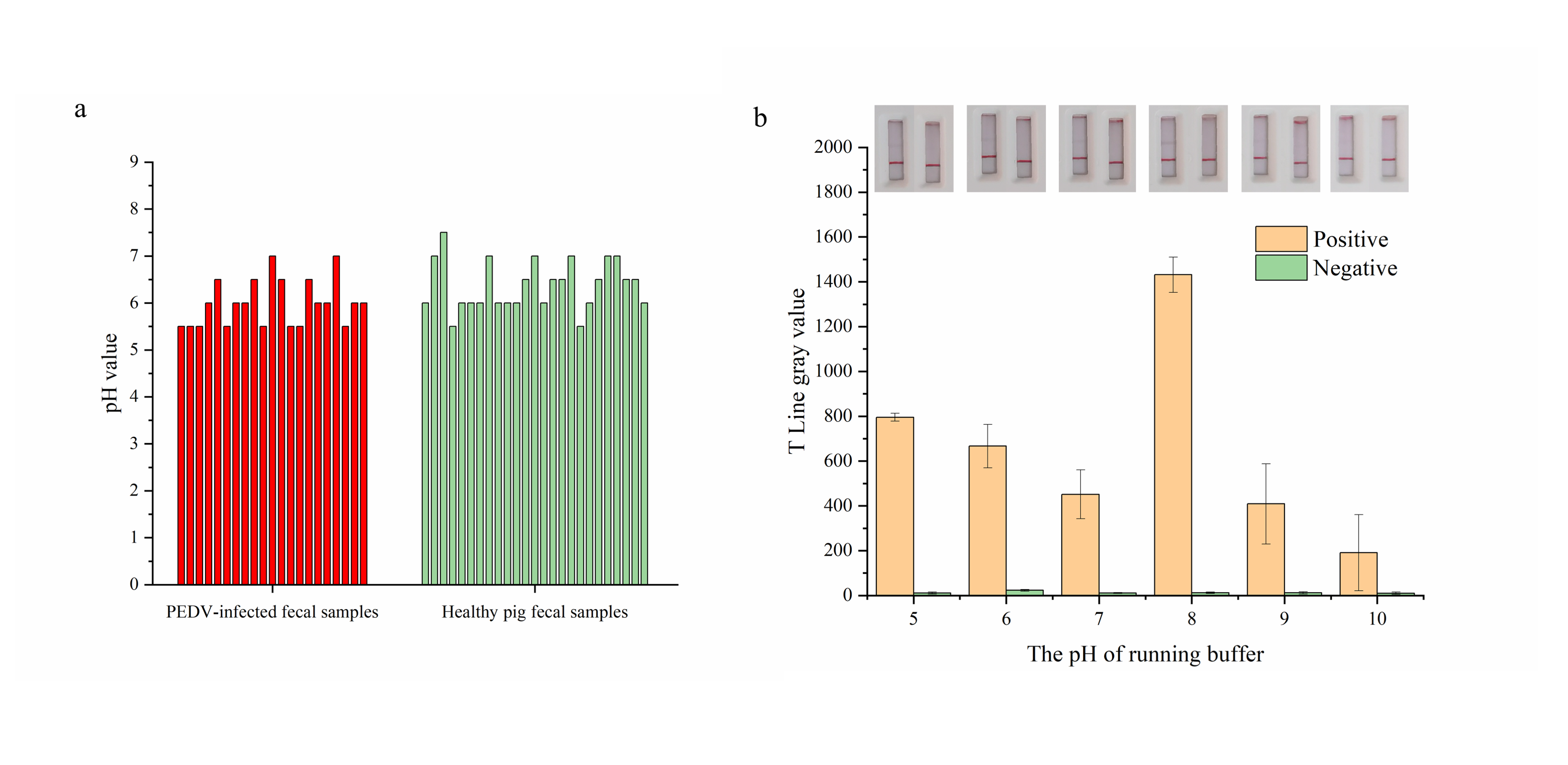

Supplement: Supplementary file 1 — Additional file 1. [file 44149_2021_29_MOESM1_ESM.zip › Fig S4.tif.tif]

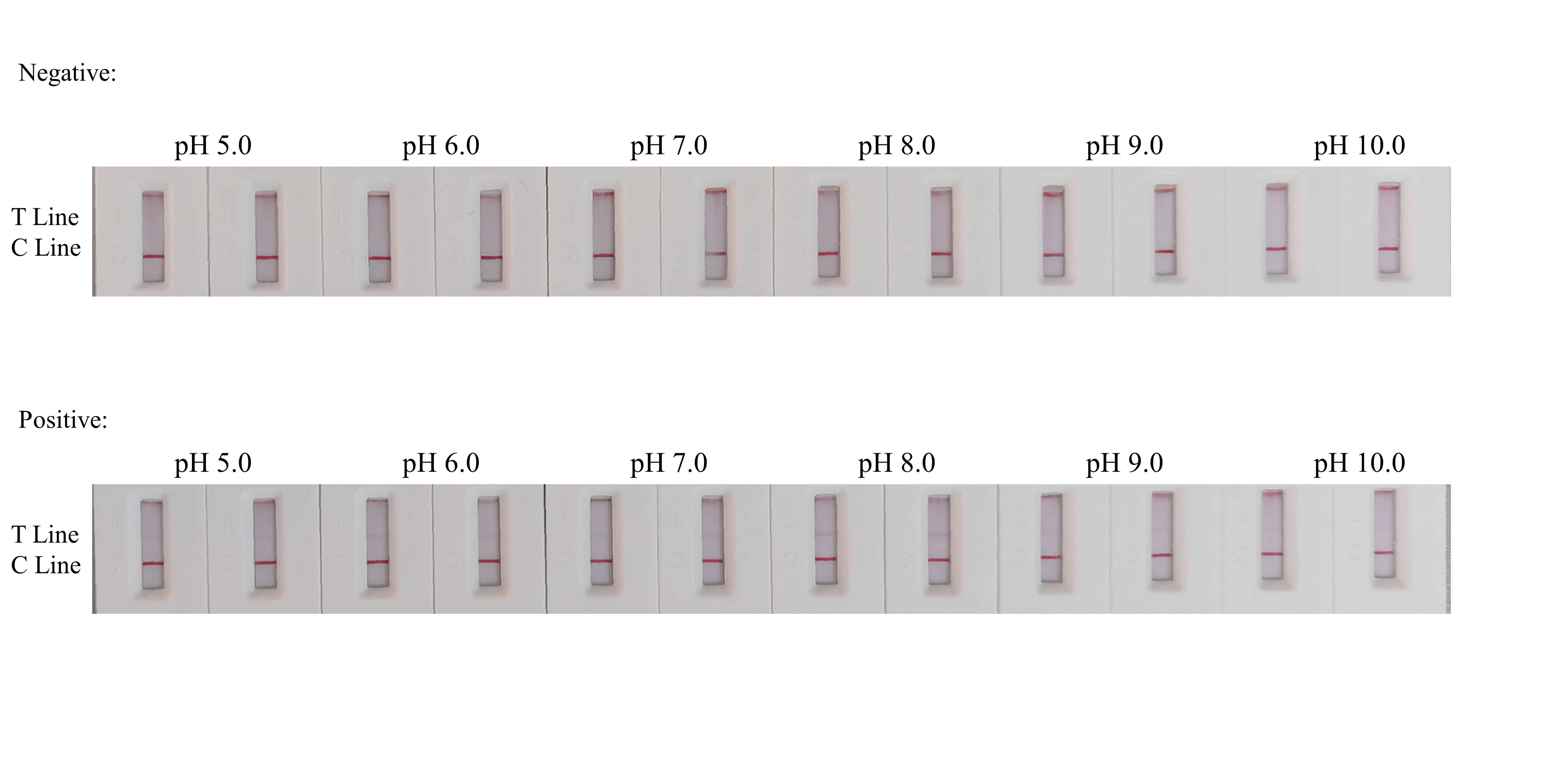

Supplement: Supplementary file 1 — Additional file 1. [file 44149_2021_29_MOESM1_ESM.zip › Fig S5.tif.tif]

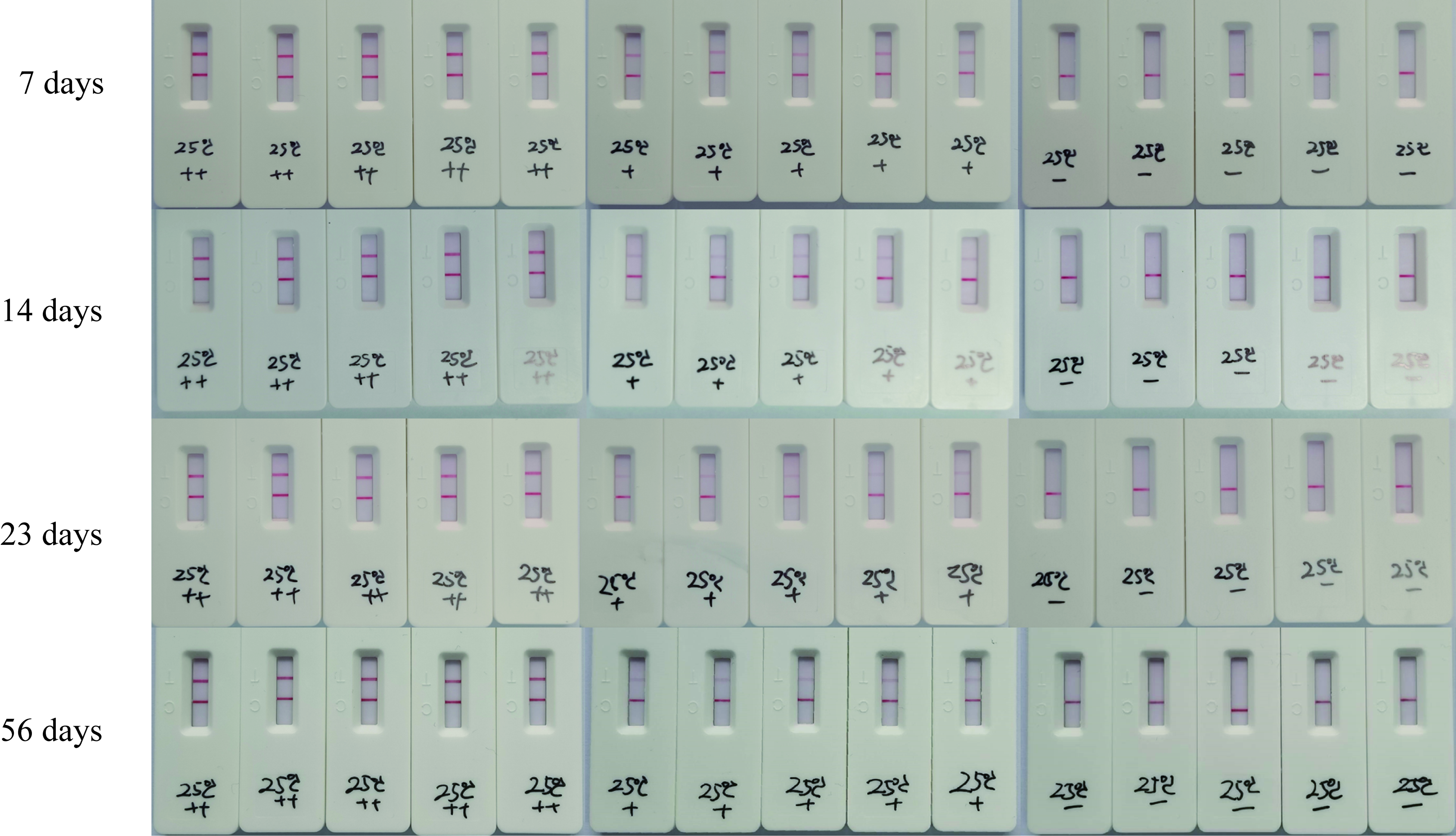

Supplement: Supplementary file 1 — Additional file 1. [file 44149_2021_29_MOESM1_ESM.zip › Fig S7.tif.tif]

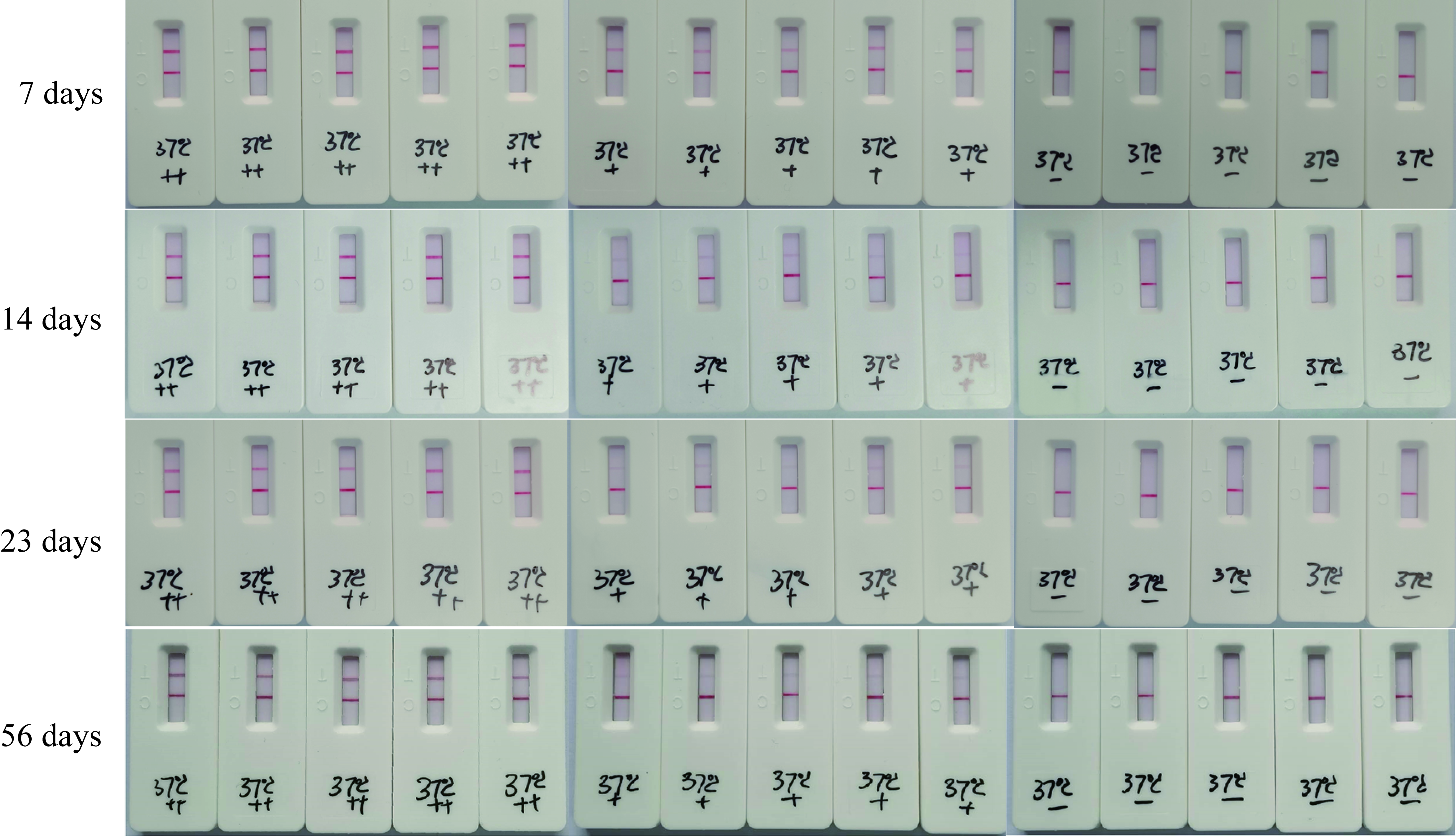

Supplement: Supplementary file 1 — Additional file 1. [file 44149_2021_29_MOESM1_ESM.zip › Fig S8.tif.tif]
